# Quality of life of people with diabetes mellitus pre revascularization surgery and post amputation: an exploratory study

**DOI:** 10.1186/1758-5996-7-S1-A19

**Published:** 2015-11-11

**Authors:** Ligia de Loiola Cisneros, Raquel Luiza Lopes Teixeira, Tulio Pinho Navarro

**Affiliations:** 1 Universidade Federal de Minas Gerais, Belo Horizonte, Brazil

## Background

Diabetic foot complications are the most serious and costly problem of Diabetes Mellitus affecting substantially the quality of life of these patients. People with diabetic foot ulcers experience severe restrictions on daily life as a result of the ulcer. They face social isolation due to reduced mobility, they require frequent clinical treatment and constant caution to ensure that effective care is taken of the feet.

## Objective

The primary aim of this study was to compare the patient perceptions of their quality of life in two moments of the timeline of the diabetic vascular disease: pre revascularization surgery period and post amputation.

## Materials and methods

An exploratory cross-sectional study was done in a sample of 28 patients with peripheral vascular complications of diabetes, divided in two groups of 14 individuals. One group consisted of outclinic patients with lower limb amputation (transtibial or transfemoral level) for at least six months. Another group consisted of hospitalized patients with critical ischemia of the lower limbs, waiting for revascularization procedures. The Portuguese version of the Medical Outcomes Study 36 -Item Short Form (SF- 36) and the Brazilian version of the Problems Areas in Diabetes Scale (B-PAID) questionnaires were used.

## Results

Patients who had been through amputation had significant (p< 0.05) lower impairment in the total score (46.48±24.91; 30.74±11.0), vitality (80.00±29.70; 47.50±18.05), role emotional (50.00±44.54; 31.25±29.60) and mental health (61.14±27.10; 36.86±18.17) domains than pre revascularized patients. The statistical difference between groups regarding the B-PAID questionnaire score was significant (p<0.01), with the best Results showed by patients with amputation (44.57±18.23; 70.93±19.72).

## Conclusion

Those findings demonstrate that diabetic patients with critical ischemia in the lower limbs had worst quality of life scores than those with amputation. The postponing of an amputation must be well judged when a critical ischemia of the lower limbs is seriously impairing the patient's quality of life.

